# Carbon monoxide pollution aggravates ischemic heart failure through oxidative stress pathway

**DOI:** 10.1038/srep39715

**Published:** 2017-01-03

**Authors:** Cyril Reboul, Julien Boissière, Lucas André, Gregory Meyer, Patrice Bideaux, Gilles Fouret, Christine Feillet-Coudray, Philippe Obert, Alain Lacampagne, Jérôme Thireau, Olivier Cazorla, Sylvain Richard

**Affiliations:** 1 LAPEC EA4278, Université Avignon, Avignon, France; 2 INSERM U1046, CNRS UMR 9214, Université Montpellier, Montpellier, France; 3 INRA UMR 866, Unité Dynamique Musculaire et Métabolisme, Montpellier, France

## Abstract

Risk of hospital readmission and cardiac mortality increases with atmospheric pollution for patients with heart failure. The underlying mechanisms are unclear. Carbon monoxide (CO) a ubiquitous environmental pollutant could be involved. We explored the effect of daily exposure of CO relevant to urban pollution on post-myocardial infarcted animals. Rats with ischemic heart failure were exposed 4 weeks to daily peaks of CO mimicking urban exposure or to standard filtered air. CO exposure worsened cardiac contractile dysfunction evaluated by echocardiography and at the cardiomyocyte level. In line with clinical reports, the animals exposed to CO also exhibited a severe arrhythmogenic phenotype with numerous sustained ventricular tachycardias as monitored by surface telemetric electrocardiograms. CO did not affect cardiac β–adrenergic responsiveness. Instead, mitochondrial dysfunction was exacerbated leading to additional oxidative stress and Ca^2+^ cycling alterations. This was reversed following acute antioxidant treatment of cardiomyocytes with N-acetylcysteine confirming involvement of CO-induced oxidative stress. Exposure to daily peaks of CO pollution aggravated cardiac dysfunction in rats with ischemic heart failure by specifically targeting mitochondria and generating ROS-dependent alterations. This pathway may contribute to the high sensibility and vulnerability of individuals with cardiac disease to environmental outdoor air quality.

Air pollution is a general public health issue with major cardiovascular and economic consequences^1^. The World Health Organization estimates that air pollution is responsible for 3 million deaths worldwide in 2012^2^. Temporal associations have been shown between exposure to air pollutants and acute decompensated heart failure (HF) hospitalization and HF mortality^1^,^3^,^4^,^5^. Increased cardiovascular mortality following long term exposure to air pollution contributes greatly to premature death^6^. Among various environmental air pollutants, carbon monoxide (CO) is ubiquitously produced from many common sources (cigarette and second-hand smoke, vehicular exhaust, industrial emissions) and plays a critical role in pollution-induced cardiac events^7^,^8^. Daily exposure to ambient CO has been associated with increased hospital admissions, particularly for individuals with cardiovascular disease^7^,^9^. At high doses, CO is toxic by reducing oxygen carrying capacities. Instead at low level, CO acts as a gasotransmitter activating signaling pathways^10^. It has been proposed that oxidative stress could be involved at some point in the mechanisms behind morbidity and mortality related with traffic-related air pollution^11^. However, because it is difficult to separate the effects of CO from those of other confounding atmospheric pollutants in those epidemiologic studies, the mechanisms underlying specifically the toxicity of environmental CO pollution in individuals with HF are unknown.

We previously showed in experimental conditions mimicking daily urban pollution with CO that healthy rats develop a HF-like cardiac phenotype with altered intracellular Ca^2+^-handling, enhanced sympathetic nervous system activity and occurrence of arrhythmia^12^. In addition, those animals exhibited higher cardiac vulnerability to ischemia-reperfusion with augmented cell death and severe arrhythmias^13^. Thus daily exposure to CO levels consistent with urban life activates specific deleterious pathways that alter cardiac function. The present work aimed to investigate the impact of this CO-induced deleterious pathway on vulnerable animals with decompensated HF. Experiments were performed 24 hours after the last CO exposure to ensure a similar carboxyhemoglobin (HbCO) level than that of the control group. We show that CO exposure enhanced the cardiac defects associated with HF both *in vivo* and at the cardiomyocyte level by specifically targeting mitochondria and generating ROS-dependent defects.

## Results

### CO worsens cardiac dysfunction of rats with heart failure

**Figure 1:**
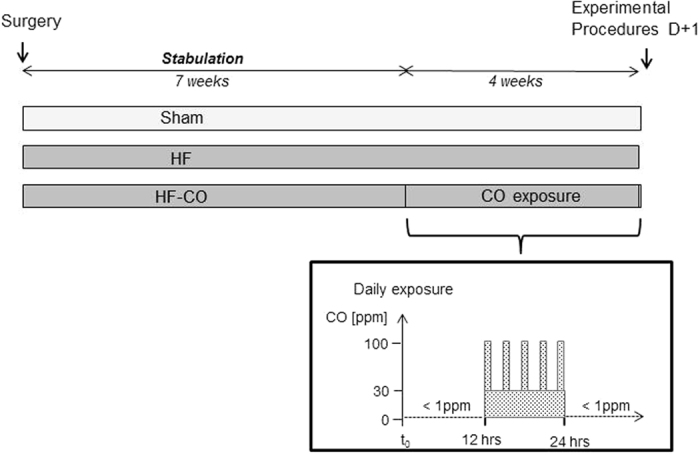
Experimental design. The three experimental groups consisted of Sham operated, non-exposed heart failure (HF) and CO-exposed heart failure (HF-CO) rats. Rats underwent (HF) or not (Sham) a tightening of the left coronary artery ligature to induce myocardial infarction. Seven weeks after coronary artery ligation, HF rats were exposed (HF-CO) or not (HF) to simulated CO air pollution 12 h/day, 7 days/week for 4 weeks.

**Figure 2:**
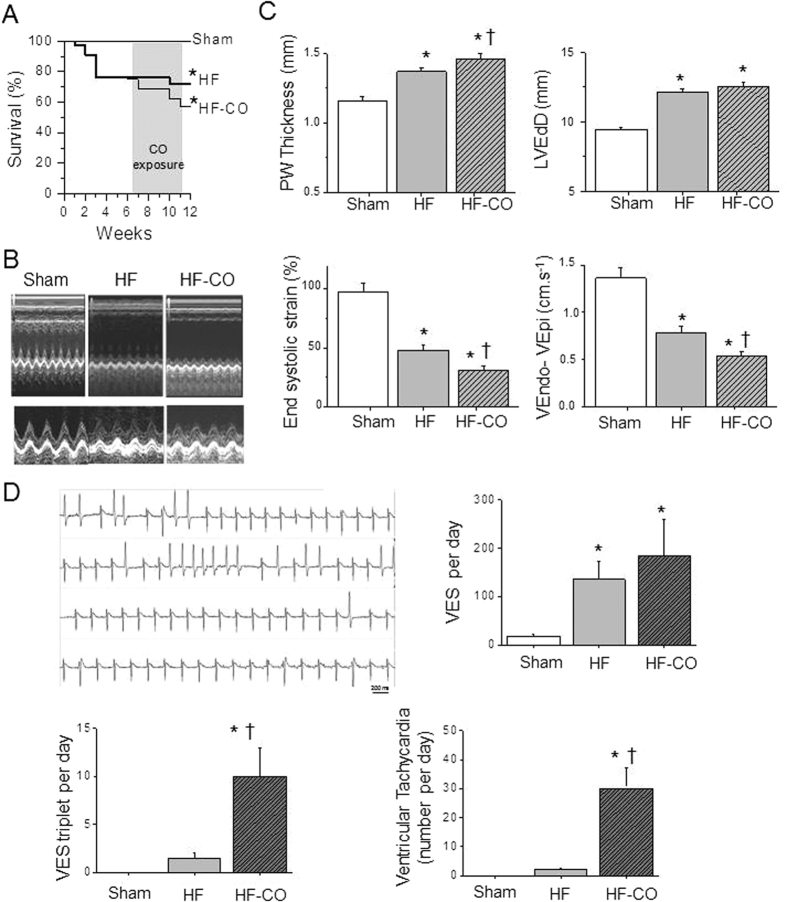
CO aggravates HF-induced LV remodeling and arrhythmic phenotype. (**A**) Survival curves of the Sham (n = 14), HF (n = 22) and HF-CO (n = 17) rats. The grey zone delineates the period of exposure to CO of the HF-CO group (*p < 0.01 *vs*. Sham; Log-rank (Mantel-Cox) test). (**B**,**C**) Echocardiographic exploration of cardiac function in Sham (n = 11), HF (n = 11) and HF-CO (n = 11) rats. (**B**) Representative TM measurements of the left ventricle (LV). (**C**) Left ventricular end-diastolic diameter (LVEdD); posterior wall thickness (PW thickness); end-systolic strain and Endo-Epi velocity gradient of the posterior wall. (**D**) Electrocardiographic exploration. Top left panel: Representative examples of ventricular ectopic beats and tachycardia in HF-CO rats. Right and lower panels: Number of isolated and triplet (three consecutive) ventricular extrasystoles (VEs), and tachycardic episodes (more than 3 VEs) during 24 hours in Sham (n = 8), HF (n = 8) and HF-CO (n = 6) rats. *p < 0.05 *vs*. Sham, ^†^*p* < 0.05 for HF-CO *vs*. HF rats; ANOVA followed by Bonferroni’s *post-hoc* tests.

Male Wistar rats (6 weeks old) were randomly assigned in three groups: Sham (n = 30), rats with heart failure (HF; n = 24) and HF rats exposed to CO (HF-CO; n = 15). In large cities, atmospheric CO varies between 2 to 40 ppm, but can peak under certain circumstances (heavy traffic, smoke environment) to 150–200 ppm^14^,^15^. To simulate daily variations of environmental CO HF rats were first exposed to filtered air (<1 ppm of CO) for 12 hours. Then, CO level was increased to a basal level of 30 ppm for 12 hours with five peaks of 100 ppm of 1 hour each (Fig. 1)^12^. Within 11 weeks post-infarct, 27% of HF rats and 41% of HF rats exposed to CO (HF-CO) died (Fig. 2A) showing the impact of HF on mortality. CO had no significant additional effect during the 4-week exposure time despite a trend for divergence between the HF and HF-CO curves at the end of the protocol. Cardiac morphology and function were evaluated by Doppler echocardiography and tissue Doppler imaging at the end of the protocol. Left coronary ligation induced morphological and functional cardiac defects (Fig. 2B,C, Table 1). Echocardiography of the left ventricle (LV) revealed an akinetic anterior wall, chamber dilation and an increased posterior wall thickness (PWT, +18% versus Sham) (Fig. 2B,C). Hypertrophy was confirmed by an increase in heart weight measured post-mortem (Table 1). LV systolic dysfunction in HF versus Sham was revealed by a reduced shortening fraction (Table 1), a decreased end-systolic strain of the posterior wall (−50%), and a decreased Endo-Epi velocity gradient across the LV (−43%) (Fig. 2C).

Chronic CO exposure worsened the cardiac alterations of HF rats (Fig. 2B,C). Cardiac hypertrophy was augmented in the HF-CO rats when compared to HF animals as shown by a greater increase in posterior wall thickness (+26% versus Sham) and heart weight (Table 1; Fig. 2B,C). Hypertrophy was also characterized by more severe regional myocardial contractile dysfunction in HF-CO versus Sham as indicated by the greater decreases in LV posterior wall end-systolic strain (−70%) and the Endo-Epi velocity gradient (−61%) in the HF-CO group compared to the HF group (Fig. 2C). This aggravation of contractile defects and hypertrophy following CO exposure in HF-group was associated with an amplified arrhythmic phenotype as revealed by telemetric electrocardiogram (ECG) recordings (Fig. 2D). Indeed, HF-CO rats developed more frequent ventricular extrasystoles than HF rats (Fig. 2D). Moreover, the occurrence of sustained ventricular tachycardia, absent in Sham and very low in HF rats (≈2 events/day), was greatly exacerbated in HF-CO rats (≈30 events/day). Taken together, these data suggest that chronic CO exposure aggravates morbid cardiac phenotype and dysfunction in rats with established HF, which could potentially lead to the premature death in HF-CO animals. CO-dependent alterations of excitation-contraction coupling and Ca^2+^ signaling within the cardiomyocytes may explain this additional effect of CO exposure both on contractile and rhythmic functions^12^.

### CO worsens defective excitation-contraction coupling of failing hearts

**Figure 3:**
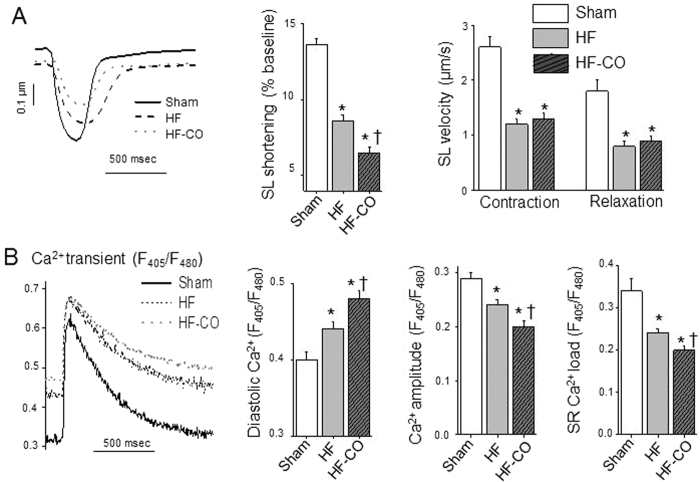
CO provokes contractile dysfunction in HF rats and alters the Ca^2+^ dynamics in ventricular myocytes of HF rats. (**A**) Left panel: Examples of cell shortening in myocytes isolated from Sham (line), HF (black dashed line) and HF-CO (grey dotted line) rats paced at 0.5 Hz. Averaged amplitude of sarcomere length shortening (middle panel) and velocities of contraction and relaxation (right panel) of Sham (n = 53 cells from 4 rats), HF (n = 104 cells from 5 rats) and HF-CO (n = 68 cells from 3 rats) myocytes. (**B**) Left panel: Superimposed examples of Ca^2+^ transients (left) and diastolic intracellular Ca^2+^(right) in intact isolated myocytes from Sham (line, white bar), HF (black dashed line, gray bar) and HF-CO (grey dotted line, hatched bar) rats. Averaged diastolic Ca^2+^ (middle panel) and, Ca^2+^-transient amplitude (right panel) of Sham (n = 53 cells from 4 rats), HF (n = 104 cells from 5 rats) and HF-CO (n = 68 cells from 3 rats) myocytes. SR Ca^2+^ load was determined after caffeine application in Sham (n = 9 cells from 3 rats), HF (n = 12 cells from 4 rats) and HF-CO (n = 16 cells from 4 rats) myocytes. *p < 0.05 *vs*. Sham, ^†^*p* < 0.05 HF-CO *vs*. HF; ANOVA followed by Bonferroni’s post-hoc tests.

Single LV myocytes obtained following enzymatic dissociation at the end of the protocol were used to measure simultaneously cell shortening (an index of contractility) and cytoplasmic Ca^2+^ content indexed by the ratiometric fluorescent indicator Indo-1. Intact LV myocytes of HF rats displayed impaired contractility and relaxation when compared to Sham (Fig. 3A). Cell shortening was decreased in HF myocytes and further declined in HF-CO myocytes (−37% and *−*52% *versus* Sham, respectively). The kinetics of contraction and relaxation already decreased in HF myocytes were not further altered by CO. Myocyte contractility depends both on the amount of Ca^2+^ released from the sarcoplasmic reticulum (SR) and on the Ca^2+^ sensitivity of myofilaments.

**Figure 4:**
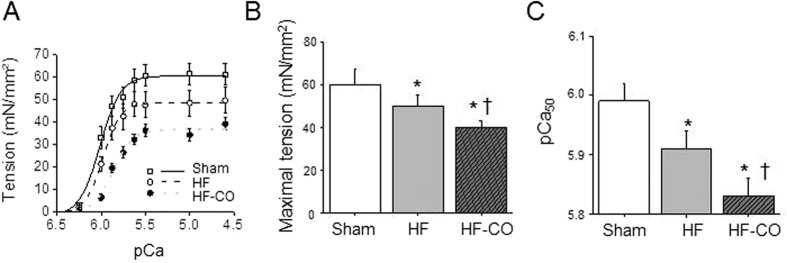
Chronic CO pollution alters the contractile machinery in permeabilized cardiomyocytes of HF rats. (**A**) Averaged tension-pCa curves of permeabilized cardiomyocytes. (**B**) Maximal active tension and (**C**) myofilament Ca^2+^ sensitivity (pCa_50_) were calculated from in Sham (n = 14 cells from 4 rats), HF (n = 19 cells from 5 rats) and HF-CO (n = 12 cells from 3 rats) permeabilized myocytes. *p < 0.05 *vs*. Sham, ^†^*p* < 0.05 HF-CO *vs*. HF; ANOVA followed by Bonferroni’s post-hoc tests.

Therefore, we evaluated Ca^2+^ homeostasis in cells loaded with the Indo-1 signal and stimulated at 0.5 Hz (Fig. 3B). HF cardiomyocytes exhibited Ca^2+^-handling disruptions including increased diastolic Ca^2+^, as reflected by the increase of baseline Ca^2+^ level, and a decrease of the Ca^2+^ transient amplitude indexed by the difference between the peak and the baseline Ca^2+^ value, when compared to Sham animals (Fig. 3B). The Ca^2+^-reuptake time constant (tau) measured from the exponential fit of the Ca^2+^ transient decay was increased in HF cells (0.45 ± 0.01 s) *versus* Sham cells (0.36 ± 0.02 s). The amplitude of rapid Ca^2+^ release following caffeine application that reflects SR Ca^2+^ load was also reduced in HF rats *versus* Shams (Fig. 3B). Chronic exposure to CO in HF rats exacerbated the cellular diastolic Ca^2+^ overload, and the reductions of the Ca^2+^-transient amplitude and SR Ca^2+^ load observed in HF rats. The Ca^2+^-reuptake time constant was not different between the HF and HF-CO cells (0.46 ± 0.02 s). Next, we investigated specifically the properties of the contractile machinery by measuring in permeabilized LV myocytes the relationship between the Ca^2+^-activated force and the intracellular free Ca^2+^ concentration (Fig. 4A). The maximal isometric tension normalized to cross-sectional area was decreased in HF rats and further decreased in HF-CO animals (−17% and −33% *versus* Sham, respectively) (Fig. 4A). Moreover, the tension-pCa curve shifted to the right in HF rats (*i.e*. decrease in pCa_50_) indicating a desensitization of myofilaments to Ca^2+^ and further in HF-CO myocytes (Fig. 4A). Altogether, we observed CO-induced alterations in cellular contractility both at the level of Ca^2+^ homeostasis and of the contractile machinery. Those changes may contribute to the reduced contractile function and arrhythmic events observed *in vivo*.

### CO pollution does not modify cardiac β-adrenergic reserve in HF rats

**Figure 5:**
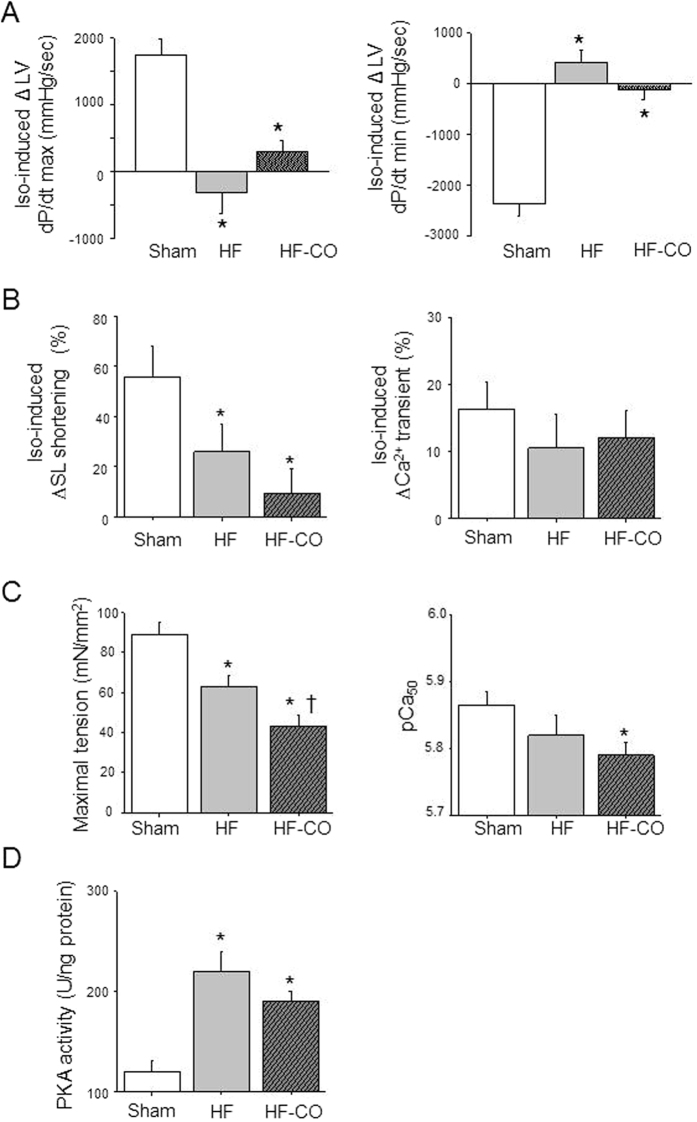
Effect of CO pollution on the cardiac β-adrenergic reserve in HF rats. (**A**) Peak rate variation of the first derivative of LV pressure rise and fall (dP/dt max and dP/dt min, respectively) during isoproterenol perfusion (1 mg kg^−1 ^min^−1^; Iso-induced) evaluated *in vivo* (n = 9 rats/group). (**B**) Variation of sarcomere length shortening (SL) and Ca^2+^ transients in isolated LV cells in presence of isoproterenol (100 nmol/L; Iso-induced) from in Sham (n = 14 cells from 5 rats), HF (n = 15 cells from 5 rats) and HF-CO (n = 9 cells from 3 rats) intact myocytes. (**C**) Maximal active tension (left) and myofilament Ca^2+^ sensitivity (right) of permeabilized cardiomyocytes after PKA treatment (100 U mL^−1^) from in Sham (n = 15 cells from 4 rats), HF (n = 7 cells from 4 rats) and HF-CO (n = 12 cells from 4 rats) permeabilized myocytes. (**D**) PKA activity measured in cardiac tissue (n = 4 hearts). *p < 0.05 *vs*. Sham, ^†^*p* < 0.05 HF-CO *vs*. HF; ANOVA followed by Bonferroni’s post-hoc tests.

Modification of the adrenergic signaling pathway due to chronic activation of sympathetic nervous system is commonly observed in hypertrophy leading to contractile and rhythmic disturbances^16^. Meantime, we previously showed that chronic CO exposure activates the cardiac sympathetic system (i.e. the β-adrenergic pathway) and decreases cardiac β-adrenergic reserve in healthy rats^12^. Thus, we explored the impact of CO on the cardiac response to β-adrenergic stimulation of rats with HF both *in vivo* and *in vitro*. In Sham animals, the β-adrenergic agonist isoproterenol increased *in vivo* cardiac contractility, as indicated by the higher LV developed pressure (Table S1) and the maximal rate of change of LV pressure during contraction (dP/dtmax) and relaxation (dP/dtmin) (Fig. 5A). These positive inotropic and lusitropic effects induced by isoproterenol were blunted in HF rats *versus* Sham rats (Fig. 5A) without further aggravation in HF-CO rats. Isoproterenol increased cell shortening (Fig. 5B, left panel). This effect was smaller in HF cardiomyocytes than in Sham (+20% *vs*. +60%, respectively) and was unchanged by chronic CO exposure. The potentiating effect of isoproterenol on the Ca^2+^-transient amplitude was similar in Sham and HF myocytes (Fig. 5B, right panel) and was unaffected by CO exposure, suggesting that alterations of the myofilament response could underlie the reduced contraction of HF myocytes.

We next explored whether maximal β-adrenergic stimulation could normalize the properties of the myofilaments in HF rats. In the cardiomyocyte, β-adrenergic stimulation activates protein kinase A (PKA), which phosphorylates and potentiates activities of proteins involved in excitation-contraction coupling^17^. We then deciphered whether a differential level of PKA stimulation can explain the difference of myofilament Ca^2+^ sensitivity. For this purpose, permeabilized myocytes were incubated with a recombinant catalytic subunit of PKA (Fig. 5C). After PKA treatment, the maximal tension remained lower in HF myocytes than in Sham and even lower in HF-CO myocytes (Fig. 5C). PKA, which induces the Ca^2+^ desensitization of myofilaments by phosphorylating sarcomeric proteins, decreased the pCa_50_ in all groups of animals (Fig. 5C). The difference in myofilament Ca^2+^ sensitivity between Sham and HF as well between HF and HF-CO myocytes observed under baseline conditions (Fig. 3B) disappeared after PKA treatment. However, the difference between Sham and HF-CO myocytes was still observed (p < 0.01). Together, these results indicate that differential PKA activation levels cannot explain the changes observed after CO exposure. In addition, PKA activity was increased in HF rats, and CO exposure did not alter this level (Fig. 5D). Thus, the altered β-adrenergic pathway in HF animals was not further affected by chronic exposure to CO. Urban pollution is known to favor oxidative damages^18^. Thus, we assessed if modifications of oxidative status induced by CO could be responsible for the increase in cardiac dysfunction observed in HF-CO animals.

### CO pollution alters mitochondrial function and increases ROS production

**Figure 6:**
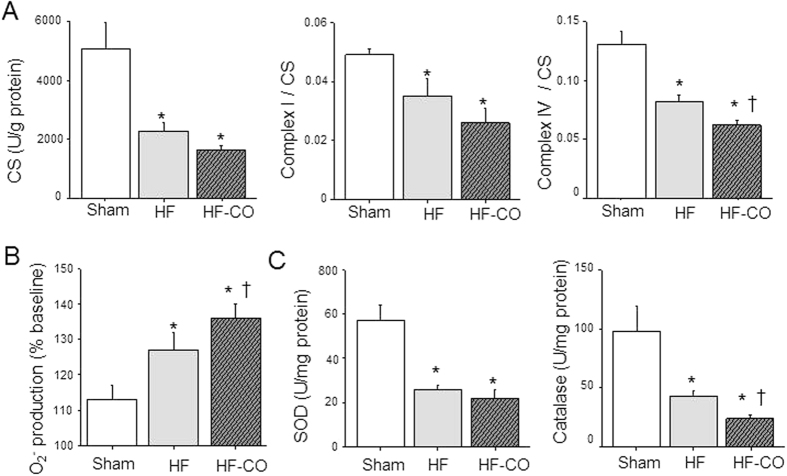
Oxidative stress is responsible for CO-associated defects in HF rats. (**A**) Activities of citrate synthase (CS, left), and complexes I (middle) and IV (right) of the electron transport chain in cardiomyocytes (n = 8 hearts). (**B**) Production of O_2_^−^ was measured using MitoSOX Red fluorescence in cardiomyocytes paced at 0.5 Hz in Sham (n = 22 cells from 4 rats), HF (n = 45 cells from 6 rats) and HF-CO (n = 32 cells from 4 rats) intact myocytes. (**C**) Activities of the antioxidant enzymes SOD and catalase in cardiomyocytes (n = 8 rats per group). *p < 0.05 *vs*. Sham, ^†^*p* < 0.05 HF-CO *vs*. HF; ANOVA followed by Bonferroni’s post-hoc tests.

Mitochondria are important sites of ROS production during chronic disease due to deficits in the electron transport chain^19^. It is documented that CO binding to cytochrome c oxidase (also named Complex IV) in the electron transport chain leads to the generation of reactive oxygen species (ROS)^20^. We assessed in cardiomyocytes the activities of citrate synthase (CS) an index of mitochondrial oxidative metabolism^21^ and/or of mitochondria content^22^, and the activities of complex I and IV of the electron transport chain (Fig. 6A). CS activity was decreased in HF rats by 56% *versus* Sham. Although the difference between HF and HF-CO cells did not reach significance (p = 0.08), CS activity was further decreased after CO exposure (by −67% *versus* Sham). The activities of both complexes I and IV were reduced in HF myocytes *versus* Sham by −37% and −29%, respectively. CO exposure did not further decrease complex I activity (−53% *versus* Sham) but exacerbated the deficit in complex IV activity in HF rats (−46% *versus* Sham) (Fig. 6A). The altered mitochondrial metabolism in HF rats by CO may affect ROS production. We next measured the mitochondrial production of superoxide anions (O_2_^−^) in myocytes using MitoSOX Red^23^. After 5 minutes of contraction at 1 Hz, myocytes from HF rats produced about 12% more mitochondrial ROS than Sham myocytes, while HF-CO myocytes produced about 23% more ROS (Fig. 6B). Since oxidative stress results from an imbalance between pro-oxidant activities and antioxidant defenses that buffer the ROS, we evaluated the activity of two key antioxidant enzymes in cardiomyocytes: superoxide dismutase (SOD), which converts O_2_^−^ to H_2_O_2_, and catalase, which converts H_2_O_2_ to H_2_O. Both SOD and catalase activities were reduced in HF rats compared to Sham. CO exposure did not further modify SOD activity but decreased catalase activity to a higher extent (Fig. 6C). Taken together, the results indicate that the pro-oxidative stress status in HF rats is aggravated by CO exposure.

**Figure 7:**
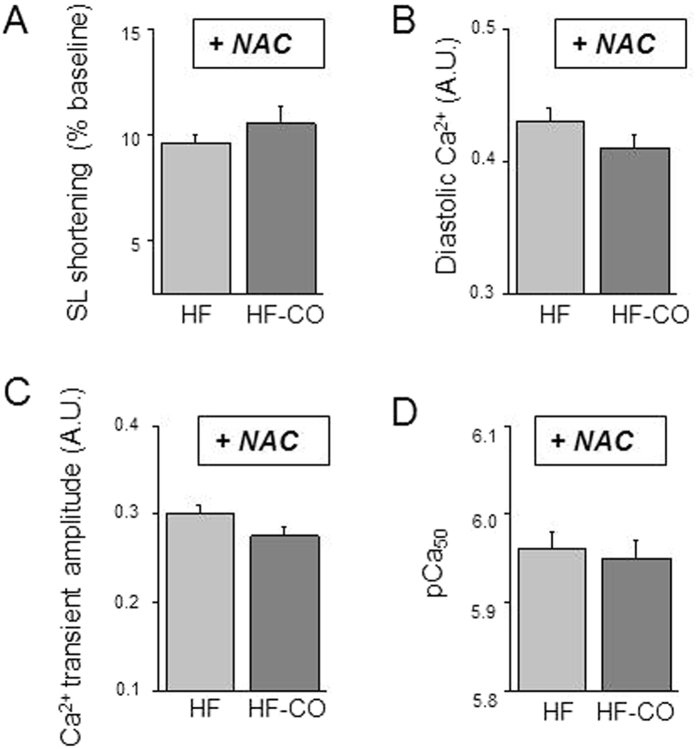
Effects of antioxidant treatment (NAC) on cell contractile function. (**A**–**C**) Averaged sarcomere shortening (SL shortening) (**A**), diastolic intracellular Ca^2+^ (**B**), and Ca^2+^-transient amplitude (**C**) after NAC treatment in HF (n = 54 cells from 5 rats) and HF-CO (n = 21 cells from 4 rats) intact myocytes (n = 31–54 cells/4–5 hearts/group). (**D**) Myofilament Ca^2+^ sensitivity (pCa_50_) of myocytes treated with NAC then permeabilized in HF (n = 9 cells from 4 rats) and HF-CO (n = 11 cells from 4 rats) intact myocytes. *p < 0.05 *vs*. HF; ANOVA followed by Bonferroni’s post-hoc tests.

As a proof of concept of the involvement of ROS in the CO-induced specific defects of cell contractility, cardiomyocytes were incubated with the large-spectrum antioxidant NAC (20 mmol/L) for 1 hour. Antioxidant treatment erased the difference of sarcomere shortening between HF and HF-CO myocytes (Fig. 7A). Similarly, NAC treatment abolished the differences in diastolic Ca^2+^ (Fig. 7B), systolic Ca^2+^ release (Fig. 7C), and myofilament Ca^2+^ sensitivity (Fig. 7D) between HF and HF-CO myocytes, and restored these to Sham levels. Our results indicate that the additional deleterious effects of chronic low-level CO exposure in HF are mediated by ROS-dependent mechanisms.

## Discussion

Epidemiologic studies suggest that environmental pollution is associated with cardiovascular outcomes and can cause premature death, notably in vulnerable populations. The mechanisms underlying these effects are poorly understood. In particular, many research groups currently focus only on fine particulate pollutants and CO is less and less studied. Nevertheless, CO is one of six pollutants for which the American Environmental Protection Agency has established National Ambient Air Quality Standards^15^. The present study showed that 4-week exposure under controlled conditions mimicking daily urban environmental pollution to CO worsened the progression of HF following myocardial infarction.

We demonstrate here in an animal model that chronic CO exposure aggravates contractile dysfunctions in HF, both *in vivo* and at the cellular levels, and enhanced the susceptibility to potentially lethal arrhythmia. During HF, the β-adrenergic pathway is hyperactivated^23^ and participates to cellular dysfunction, in particular for the diastolic and systolic Ca^2+^ defects mediated by phosphorylation of proteins involved in Ca^2+^ cycling^24^,^25^. These alterations could result from altered expression and/or function of both SERCA2a and type 2 ryanodine receptor following chronic adrenergic stimulation^16^. Here, we showed that the lower contractility in CO exposed HF rats was unrelated to a difference in β-adrenergic activation. Indeed, the response to isoproterenol challenge was similarly blunted in HF rats with or without exposure to CO. Conversely, we showed that CO exposure amplifies morbid effect of ischemic HF via uncontrolled ROS production and additionally altered Ca^2+^ cycling. ROS are potent molecules affecting reversibly or irreversibly Ca^2+^ signaling proteins^26^.

Energy and oxidative metabolism are altered in HF^27^ leading to exacerbated ROS production^28^ Excess of ROS targets proteins involved in Ca^2+^ homeostasis^29^ and in the contractile machinery^23^, which contributes to HF-associated contractile deficits^28^. Here, we showed that CO exposure decreases cytochrome c oxidase (complex IV) activity and increases ROS production by the mitochondria. This is in line with previous reports showing that CO binds to the ferrous heme of complex IV, which inhibits the electron transport chain^30^, induces electron accumulation and O_2_^−^ production at the complex III level, accounting for most of the O_2_^−^ produced in the heart^31^. Endogeneous CO via NADPH oxidase 4 (Nox4) can produce mitochondrial ROS. We did not explore the role of Nox4 in the context of exogenous exposure of CO. Indeed, the consequences of Nox4 activation on the cardiovascular function are still controversial^32^ and several works described cardioprotective effects^32^,^33^. Its potential contribution in our model warrants further experiments. Moreover, it is also worth to consider that elevation of ROS is due to the interaction of CO with antioxidant enzymes such as SOD and catalase. Indeed, catalase is a heme-containing enzyme that can interact with CO, potentially affecting its activity^34^. This mechanism could explain the lower catalase activity in HF-CO rats observed in our study while SOD is unaffected. The link between increased O_2_^−^ production and CO-induced defects in contractility is demonstrated by the partial restoration of contractility in isolated cardiomyocytes by acute antioxidant treatment. This is consistent with previous publications showing that acute NAC treatment can scavenge excess ROS and normalize Ca^2+^ homeostasis and myofilament properties, thus preventing Ca^2+^-dependent arrhythmias in both rats with HF^23^ and healthy rats exposed to chronic low-level CO^35^. However, NAC is a large spectrum antioxidant that did not only target mitochondria-dependent ROS production. So, we cannot exclude that CO induces also non-mitochondrial ROS production, in particular via heme-containing enzymes such as nitric oxide synthases.

Like other signaling pathways, there may be a balance between the beneficial and detrimental effects of CO depending on the dose, duration of exposure and biological environment^10^. As a gas, CO can easily penetrate tissues and cells to interact with cellular proteins. Unlike nitric oxide, it is not metabolized in mammals and has to be eliminated by the lungs, which can result in relatively long-lasting effects. High concentrations of CO are clearly toxic, through compromised oxygen transport. Steady release of low doses of CO is proposed for therapeutic purpose, particularly in vascular injury following organ transplantation, pulmonary hypertension, and ischemia–reperfusion damage^36^,^10^. Nevertheless, the potential toxic effects of “nontoxic” doses of CO (30 ppm/d on average) on cardiac function reported in our study warrant further investigations of chronic treatment with low-dose CO gas inhalation.

It is now clear that urban air pollution can trigger acute myocardial infarction^37^. Nowadays, most of studies focused on the role of fine particles due to their ability to penetrate deep into the lungs and blood streams. Our study highlights that CO alone may also contribute to the pollution-induced cardiovascular outcomes, especially in populations with fragile health. This is in line with a meta-analysis from 9 million people in the U.S., which has established a correlation between exposure to atmospheric CO (separate from other polluting gases) and increased risk of hospitalization or death due to cardiovascular disorders, with an increase of at least 3.52% in the latter per 1ppm increase in CO (95% CI 2.52–4.54)^1^. It should be kept in mind that combined effects with that of other gaseous or fine particle pollutants may enhance the impact. Meantime, patients with cardiomyopathy often have metabolic comorbidities that in turn aggravate their prognosis. Since CO exposure also promotes metabolic disturbances, it should be taken into consideration in the evaluation of cardiovascular risk, especially for patients in secondary prevention. CO can have serious adverse effects even at low doses in individuals exposed repeatedly to atmospheric pollution and/or secondhand smoke^38^,^39^. Since ROS are centrally involved in the deleterious effects of CO pollution, antioxidant strategies known to increase antioxidant capacities such as supplementation therapy or exercise training in a healthy environment could provide protection against these effects^40^. In addition, the use of ß-blockers reported to combine anti-adrenergic and antioxidant properties, such as carvedilol^41^, could be a potentially interesting concept to improve protection of HF patients in a polluted environment.

## Methods

### Ethics Statement

All procedures conformed to European Parliament Directive 2010/63/EU and the 22 September 2010 Council on the protection of animals. Animal procedures were performed by authorized experimenters for *in vivo* approaches (agreement N° A 34–485, I-UnivMonttp-F1-06) and for experimental surgery (R-63UnivPASCAL-CHir1-09), in an establishment certified by the Departmental Directorate of protecting populations and animal health (N°A34-172-38). Local Ethics committee for animal welfare of Laboratory INSERM U1046 (SBEA) is in charge to follow that none animal achieve experimental endpoints according to FELASA Recommendations (guidelines of the Federation of Laboratory Animal Science Associations, http://www.felasa.eu/recommendations). The protocols were approved by the local ethics committee rules *Comité d’éthique pour l’expérimentation animale Languedoc-Roussillon* (N° CEEA-LR-12083). An expanded *Methods* section is presented in the Supplemental materials.

### Experimental design

HF animals were obtained by a permanent ligation of the left coronary artery as described previously^42^. In brief, rats were anesthetized (85 mg/kg ketamine and 5.5 mg/kg xylazine, IP) and ventilated. Sham-operated animals were subjected to the same surgical operation but without tightening the coronary ligature. Seven weeks after ligature, HF-CO group was exposed for 4 weeks to CO using an air controlled chamber as previously described^12^,^43^. HbCO levels were relatively low at the end of the exposure period with 6.1 ± 1.0%. One day after the last CO exposure, HbCO level was 1.2 ± 0.4%, value similar to HbCO measured in HF rats non-exposed to CO (1.1 ± 0.3%). Animals were euthanized by cervical dislocation. At the end of the protocol (*i.e*. 11 weeks after ligature), HF-CO rats were compared to HF rats exposed to standard filtered air and Sham animals served as control (Fig. 1).

#### *In vivo* characterization

Doppler-echocardiography was performed in anesthetized animals (50 to 75 mg/kg ketamine and 10 to 15 mg/kg xylazine, IP) with the use of a MyLab 30 ultrasound apparatus (ESAOTE, Italy) equipped with a high frequency transducer (10–12 MHz). Intraventricular pressure was measured under anesthesia with a micromanometer-tipped catheter (SPR407, Millar Instruments) before and after isoproterenol perfusion (1 mg. kg^−1^ min^−1^, i.v.). ECG were recorded by telemetry as previously described^12^.

#### Cell characterization

Single LV myocytes were isolated by enzymatic digestion at the end of the protocol. Unloaded cell shortening and Ca^2+^ variations were measured using field stimulation (0.5 Hz, 22 °C, 1.8 mM external Ca^2+^). LV cardiomyocytes were loaded with Indo1-AM 5 μM for 30 min at room temperature. Sarcomere length (SL) and fluorescence (405 and 480 nm) were simultaneously recorded (IonOptix system, Hilton, USA) in intact myocytes before and after isoproterenol (100 nmol/L)^12^,^42^. Some intact cardiomyocytes were treated with N-acetylcysteine (20 mmol/L, NAC) for 1 hour prior to the experiments. Myofilament properties were determined at 2.3 μm sarcomere length in permeabilized myocytes^12^,^42^, with or without incubation with a recombinant catalytic subunit of protein kinase A (PKA, Sigma Aldrich, France) for 50 min at room temperature as described^44^.

#### Mitochondrial characterization

We measured mitochondrial O_2_^−^ production using MitoSOX Red (5 μmol/L, Invitrogen inc., France) In isolated LV cardiomyocytes during 0.5 Hz pacing^23^. The activity of citrate synthase, complex I and IV of the electron transport chain, catalase and superoxide dismutase (SOD) was measured as previously described^12^,^42^.

#### Statistical analysis

Statistical analysis was performed using *StatView 5.0* (SAS Institute, USA). Data are presented as means ± SEM. Kaplan-Meier survival curves plot of the Sham, HF and HF-CO groups of animals (n = 14, 22 and 17 animals, respectively) where the outcome is time until death (*p < 0.05 *vs*. Sham, Log-rank (Mantel-Cox) test). Effects of CO exposure and β-adrenergic challenge were analyzed using one-way factorial ANOVA or ANOVA with repeated measures depending on the variable, followed by Bonferroni’s *post-hoc* tests if appropriate. The threshold for statistical significance was defined as *p* < 0.05.

## Additional Information

**How to cite this article**: Reboul, C. *et al*. Carbon monoxide pollution aggravates ischemic heart failure through oxidative stress pathway. *Sci. Rep.* **7**, 39715; doi: 10.1038/srep39715 (2017).

**Publisher's note:** Springer Nature remains neutral with regard to jurisdictional claims in published maps and institutional affiliations.

## Supplementary Material

Supplementary Information

## Figures and Tables

**Table 1:** Effects of chronic CO exposure on morphological parameters *in vivo*.

	Sham (n = 11)	HF (n = 11)	HF-CO (n = 11)
Morphological data			
Body wt (g)	521 ± 7	532 ± 14	539 ± 12
Heart wt (g)	1.87 ± 0.06	2.65 ± 0.14*	3.18 ± 0.16*,†
Heart wt/100 g body wt (g/100 g)	0.36 ± 0.01	0.47 ± 0.03*	0.62 ± 0.04*,†
Echocardiographic data			
LVEdD (mm)	9.39 ± 0.13	12.20 ± 0.21*	12.50 ± 0.28*
LVEsD (mm)	5.69 ± 0.16	10.68 ± 0.26*	11.15 ± 0.34*
Posterior wall thickness (mm)	1.16 ± 0.03	1.37 ± 0.03*	1.46 ± 0.04*,†
LV shortening fraction (%)	39.4 ± 1.3	12.6 ± 0.8*	10.9 ± 1.0*
E/A-wave velocity ratio	1.89 ± 0.08	3.81 ± 0.52*	4.70 ± 0.70*

Body wt: body weight; LVEdD: left ventricular end-diastolic diameter; LVEsD: left ventricular end-systolic diameter; E/A: early (E) to atrial (A) peak velocity waves ratio recorded from mitral inflow performed in pulsed Doppler mode. ^*^p < 0.05 vs. Sham, ^†^p < 0.05 *vs.* HF.
