# Supplementary material for: Carbon monoxide pollution aggravates ischemic heart failure through oxidative stress pathway
Source: Sci Rep. 2017 Jan 3;7:39715. doi: 10.1038/srep39715 (PMC5206643; doi:10.1038/srep39715)
Supplement: Supplementary Information [file srep39715-s1.doc]

**Supplemental Methods**

Carbon monoxide pollution aggravates ischemic heart failure through oxidative stress pathway

*Reboul et al. : Chronic CO pollution worsens heart failure*

Cyril Reboul^1^, Julien Boissière^1,2*^, Lucas André^2,*^, Gregory Meyer^1^, Patrice Bideaux^2^, Gilles Fouret^3^, Christine Feillet-Coudray^3^, Philippe Obert ^1^, Alain Lacampagne^2^, Jérôme Thireau^2^, Olivier Cazorla ^2,¥^, Sylvain Richard ^2,¥^

^1^ LAPEC EA4278, Université Avignon, Avignon, France

^2^ INSERM U1046, CNRS UMR 9214, Université Montpellier, Montpellier, France

^3^ INRA UMR 866, Unité Dynamique Musculaire et Métabolisme, Montpellier, France

# Methods.

**Ethics Statement.** All procedures conformed to European Parliament Directive 2010/63/EU and the 22 September 2010 Council on the protection of animals. Animal procedures were performed by authorized experimenters for in vivo approaches (agreement N° A 34- 485, I-UnivMonttp-F1-06) and for experimental surgery (R-63UnivPASCAL-CHir1-09), in an establishment certified by the Departmental Directorate of protecting populations and animal health (N°A34-172-38). Local Ethics committee for animal welfare of Laboratory INSERM U1046 (SBEA) is in charge to follow that none animal achieve experimental endpoints according to FELASA Recommendations (guidelines of the Federation of Laboratory Animal Science Associations, <http://www.felasa.eu/recommendations>). The protocols were approved by the local ethics committee rules Comité d’éthique pour l’expérimentation animale Languedoc-Roussillon (N° CEEA-LR-12083). An expanded *Methods* section is presented in the Supplemental materials.

**Animals and chronic CO exposure model:**

Male Wistar rats (6 weeks old) were housed in a room with a 12h/12h light-dark cycle and a temperature of 21°C, and given standard rodent chow and tap water ad libitum. They underwent a permanent ligation of the left coronary artery to induce myocardial infarction (HF rats) as previously described[^1^](#_ENREF_1), and compared to sham operated animals (Shams). Seven weeks after artery ligation, HF rats were exposed to CO (HF-CO group) in an airtight exposure container for 4 weeks, to simulate CO air pollution as previously described. Briefly, rats were exposed to filtered air (<1ppm of CO) for 12 hours, and the CO level increased for the remaining 12 hours to a basal level of 30 ppm that was additionally increased to 100 ppm for five peaks of 1 h each. HF-CO rats were compared to HF rats exposed only to standard filtered air.

## **Cardiac morphology and function in vivo:**

Morphological and functional cardiac adaptations were evaluated by Doppler echocardiography and tissue Doppler imaging as previously described. Twenty-four hours after echocardiography, the right carotid artery was cannulated with a micromanometer-tipped catheter (SPR 407, Millar Instruments) in anesthetized rats (pentobarbital sodium, 60mg.kg^-1^, i.p.). The catheter was then advanced into the left ventricle (LV) to record intraventricular pressure and its maximal rate of increase (LVdP/dt_max_) and decrease (LVdP/dt_min_). All measurements were performed before and after a β-adrenergic challenge with isoproterenol perfusion (1 mg.kg^-1^.min^-1^ IV).Analog outputs of LV pressure and heart rate were collected on an MP35 module (Biopac system, Gotela, Santa Barbara, USA).

**In vivo electrocardiogram recording and analysis:**

ECG recordings were carried out for one day starting two days after the 4-week period of exposure to CO or filtered air and then for 2 hours following isoproterenol injection (1 mg.kg^-1^, i.p.), as previously described[^2^](#_ENREF_2). Experimental procedures conformed to Lambeth conventions for the housing of animals for the determination of arrhythmic events[^4^](#_ENREF_4).

**Investigation of cardiac excitation-contraction coupling:**

Single myocytes were isolated by enzymatic digestion as previously described. Following enzymatic perfusion, the transmural infarct zone and border zone were discarded and the remaining LV was mechanically dissociated. The Ca^2+^ concentration was gradually increased to 1 mmol/L. In some cases, intact cardiomyocytes were treated with 20 mmol/L NAC for 1 hour prior to experiments.

**Ca^2+^ transient and fractional cell shortening:**

Unloaded cell shortening and Ca^2+^ variations (Indo-1 dye) were measured using field stimulation (0.5 Hz, 22°C, 1.8 mM external Ca^2+^). Sarcomere length (SL) and fluorescence (405 and 480 nm) were simultaneously recorded (IonOptix system, Hilton, USA) under basal conditions and after β-adrenergic stimulation with isoproterenol (100 nmol/L). Sarcoplasmic reticulum (SR) Ca^2+^ content was estimated by measuring the Ca^2+^ transient induced by rapid caffeine application (10 mmol/L), which instantly opens all ryanodine receptors of the SR.

**Force measurements in permeabilized cardiomyocytes:**

Isometric force was measured in single permeabilized cardiomyocytes[^5^](#_ENREF_5). Force was normalized to the cross-sectional area measured by imaging (IonOptix system, Hilton, USA). The relationship between Ca^2+^-activated force and internal Ca^2+^ concentration was measured at an SL of 2.3 µm and fitted to a modified Hill equation. In some experiments, permeabilized cardiomyocytes were incubated with a recombinant catalytic subunit of protein kinase A (PKA, Sigma Aldrich, France) for 50 min at room temperature as described[^6^](#_ENREF_6).

## **Mitochondrial function, ROS production and antioxidant enzyme activities**:

The activities of complexes I and IV of the electron transport chain were measured as previously described, by measuring the reduction of 2,6-dichlorophenolindophenol by NADH dehydrogenase at 600 nm (37°C)[^8^](#_ENREF_8) and by measuring the oxidation of reduced cytochrome c oxidase by spectrophotometry according to Wharton and Tzagoloff[^9^](#_ENREF_9), respectively. Citrate synthase activity was measured by measuring the color of 5-thio-2-nitrobenzoic acid generated from 5,5’-dithiobis-2-nitrobenzoic acid during citrate synthesis by the deacetylation of Acetyl-CoA, as described[^10^](#_ENREF_10).

Mitochondrial O_2_^.-^ production was measured as previously described[^11^](#_ENREF_11),. Briefly, isolated cardiomyocytes were loaded with MitoSOX Red (5 µmol/L, Invitrogen Inc., France). Fluorescent images were recorded using a Zeiss LSM 510 inverted confocal microscope. Cells were paced at 0.5 Hz using two platinum electrodes (20V, 1 msec). Images were collected at rest and after 5 min of stimulation. The resting fluorescence value (obtained after subtracting the background noise measured outside the cell) was used as the reference level.

After cell lysis in hypotonic buffer (phosphate buffer 8mmol/L, pH 7.4), catalase and superoxide dismutase (SOD) activity were measured as previously described.

## **Statistical analysis:**

The statistical analysis was performed using *StatView 5.0* (SAS Institute, USA). Data are presented as means ±SEM, unless otherwise specified. Effects of CO exposure, and/or β-adrenergic challenge were analyzed using one-way factorial ANOVA or ANOVA with repeated measures depending on the variable. These were followed by a Bonferroni‘s *post-hoc* test when appropriate. The threshold for statistical significance was defined as *p*<0.05.

# References

1 Ait Mou, Y., Reboul, C., Andre, L., Lacampagne, A. & Cazorla, O. Late exercise training improves non-uniformity of transmural myocardial function in rats with ischaemic heart failure. *Cardiovasc Res* **81**, 555-564, doi:10.1093/cvr/cvn229 (2009).

2 Andre, L. *et al.* Carbon Monoxide Pollution Promotes Cardiac Remodeling and Ventricular Arrhythmia in Healthy Rats. *Am J Respir Crit Care Med* **181**, 587-595, doi:doi:10.1164/rccm.200905-0794OC (2010).

3 Farah, C. *et al.* Moderate exercise prevents impaired Ca(2+) handling in heart of CO-exposed rat: implication for sensitivity to ischemia-reperfusion. *American Journal of Physiology-Heart and Circulatory Physiology* **299**, H2076-H2081, doi:10.1152/ajpheart.00835.2010 (2010).

4 Walker, M. J. *et al.* The Lambeth Conventions: guidelines for the study of arrhythmias in ischaemia infarction, and reperfusion. *Cardiovasc Res* **22**, 447-455 (1988).

5 Cazorla, O., Szilagyi, S., Le Guennec, J. Y., Vassort, G. & Lacampagne, A. Transmural stretch-dependent regulation of contractile properties in rat heart and its alteration after myocardial infarction. *Faseb J* **19**, 88-90 (2005).

6 Cazorla, O. *et al.* Length and protein kinase A modulations of myocytes in cardiac myosin binding protein C-deficient mice. *Cardiovasc Res* **69**, 370-380 (2006).

7 Andre, L. *et al.* Subendocardial increase in reactive oxygen species production affects regional contractile function in ischemic heart failure. *Antioxid Redox Signal* **18**, 1009-1020, doi:10.1089/ars.2012.4534 (2013).

8 Janssen, A. J. *et al.* Spectrophotometric assay for complex I of the respiratory chain in tissue samples and cultured fibroblasts. *Clin Chem* **53**, 729-734 (2007).

9 Tzagoloff, A. & Wharton, D. C. Studies on the Electron Transfer System. Lxii. The Reaction of Cytochrome Oxidase with Carbon Monoxide. *J Biol Chem* **240**, 2628-2633 (1965).

10 Mukherjee, A. & Srere, P. A. Purification of and mechanism studies on citrate synthase. Use of biospecific adsorption-elution techniques. *J Biol Chem* **251**, 1476-1480 (1976).

11 Andre, L. *et al.* Carbon monoxide exposure enhances arrhythmia after cardiac stress: involvement of oxidative stress. *Basic Research in Cardiology* **106**, 1235-1246, doi:10.1007/s00395-011-0211-y (2011).

12 Fauconnier, J. *et al.* Effects of palmitate on Ca(2+) handling in adult control and ob/ob cardiomyocytes: impact of mitochondrial reactive oxygen species. *Diabetes* **56**, 1136-1142, doi:doi: 10.2337/db06-0739 (2007).

**Table S1: Effects of chronic CO exposure on left ventricular hemodynamic parameters and response to isoproterenol.**

| **Hemodynamic data** | **Sham (n=5)** | **HF (n=6)** | **HF-CO (n=6)** |
| --- | --- | --- | --- |
| **Basal conditions** |  |  |  |
| LVDP (mmHg) | 118 ± 5 | 98 ± 4 * | 95 ± 3 * |
| LV dP/dt max (mmHg/s) | 5756 ± 524 | 4310 ± 188 * | 4177 ± 208 * |
| LV dP/dt min (mmHg/s) | -5462 ± 542 | -3441 ± 133 * | -3412 ± 174 * |
|  |  |  |  |
| **Isoproterenol** |  |  |  |
| LVDP (mmHg) | 131 ± 6 | 84 ± 7 * | 88 ± 5 * |
| LV dP/dt max (mmHg/s) | 6945 ± 499 | 3998 ± 134 * | 4293 ± 204 * |
| LV dP/dt min (mmHg/s) | -7248 ± 172 | -3016 ± 186 * | -3377 ± 249 * |
|  |  |  |  |

Abbreviations: LVDP, Left ventricular developed pressure, LV dP/dt max and LV dP/dt min: maximal and minimal first derivative of left ventricular pressure; *: *p*<0.05 *vs.* Sham.
